# Applications of Natural Products in Food

**DOI:** 10.3390/foods10020300

**Published:** 2021-02-02

**Authors:** Susana González-Manzano, Montserrat Dueñas

**Affiliations:** Grupo de Investigación en Polifenoles, Departamento de Química Analítica, Nutrición y Bromatología, Facultad de Farmacia, Universidad de Salamanca, E37007 Salamanca, Spain; mduenas@usal.es

The term natural products includes any substance produced by living organisms. A compound or a mixture of some of them included in the natural product can exert beneficial effects on the matrix in which they are present, i.e., food, dietary supplements or cosmetics. Among the beneficial effects described in food are organoleptic characteristic modification, extended shelf-life or improved technological characteristics. Moreover, foods can exert beneficial effects in the organism that consumes them. The growing number of scientific papers published in recent decades on the relationship between diet and the incidence of chronic diseases has highlighted the extraordinary possibilities offered by food to maintain, and even to improve, health status. It is necessary to correctly evaluate the effects that natural compounds could exert on both the food matrix and the organism.

Natural products can be extracted from the tissues of land plants, marine organisms or fermentation from microorganisms, among others. Also, food wastes can be an important source of bioactive compounds. A great diversity of compounds from different natural sources (i.e., phenolic compounds, dietary fibers, polysaccharides, vitamins, carotenoids, pigments and oils) can have a great variety of biological activities.

Therefore, a way to improve food, dietary supplements or cosmetics is to add natural substances that produce different biological activities. The aim of this Special Issue of *Foods* is to provide documents focused on the applications, evaluation of effects and activities of natural products in food, and also provide papers in which the mechanisms underlying the effects produced by the natural products are studied.

This Special Issue includes eleven papers that reported important findings from the research activities related to the applications of natural products in food, which help to increase the knowledge in this field. The study performed by Mäkinen and co-workers assesses thirteen Nordic antioxidant sources (blackcurrant, chokeberries, rosehips, buckwheat, scots pine heartwood, spruce inner bark, leaves of sea buckthorn, lingonberry, bilberry, goutweed, nettle and dandelion) using a subcritical water extraction process with the purpose of obtaining the antioxidant compounds from the most potential raw materials and to test their antioxidative effects in meat products [1]. Thus, these authors reported that the leaves of bilberry and sea buckthorn showed the highest antioxidant capacities. Therefore, these samples were selected for the development of a subcritical water extraction process. The conditions of subcritical water extraction were optimized for recovering maximal antioxidative capacities. These dried extracts obtained by subcritical water extraction were applied in chicken slices and pork sausage and their ability to prevent lipid oxidation was evaluated during 8 and 20 days of storage. The results showed that the leaves of bilberry and sea buckthorn prevented the lipid oxidation in meat products; therefore, the subcritical water extraction process could be considered as a sustainable and effective natural method for recovering antioxidative compounds from plant materials and provide new possibilities for developing healthier meat products.

The study carried out by Nezhadasab-Aghbash et al. is also included in this Special Issue. These authors reported the chemical composition of essential oils of *Satureja macrantha* at different growth stages (vegetative, flowering and fruiting stages) [2]. *Satureja macrantha* is a member of the genus *Satureja* (Lamiaceae), distributed in Asia, the Mediterranean area and America. Several studies have reported that the essential oils from genus *Satureja* have been used for the treatment of several diseases such as diarrhea, wounds, gastroenteritis and upper respiratory and urinary tract infections [3]. The main compounds identified by Gas Chromatography- Flame Ionization Detector (GC-FID) and Gas Chromatography-Mass Spectrometry (GC-MS) were carvacrol, thymol, *p*-cymene and γ-terpinene, with carvacrol being the major compound in all phenological stages studied. The antibacterial activity was also evaluated using the broth microdilution method against the foodborne pathogenic bacteria *Staphylococcus aereus* (ATCC23922), *Enterococcus faecalis* (ATCC29212) (Gram-positive), *Enterobacteraerogenes* (ATCC13046) and *Escherichia coli*. The obtained results showed that the strongest antibacterial activity against four important foodborne bacterial strains was obtained for the essential oils collected at the flowering stage, where the concentration of carvacrol and thymol was the highest. In this study, the antioxidant activity was also determined by the diphenyl-1-picrylhydrazyl (DPPH), 2,2’-azino-bis(3-ethylbenzothiazoline-6-sulfonic acid) (ABTS) and reducing power methods, showing that the highest radical scavenging activity of essential oils and reducing power was observed at the flowering stage. Thus, this study suggests that plants of *S. macrantha* could assure their potentiality to be used at an industrial level as a source of preservative agents when this plant was preferentially collected during flowering stages.

This Special Issue also includes the study performed by Rubab et al. [4], in which the antioxidant and antimicrobial activities of red cabbage using different solvents in vitro have been studied. This study reported that chloroform extract presented the strongest antimicrobial and antioxidant activities. Thus, the results were obtained by the characterization of red cabbage chloroform extract, carried out by GC-MS analysis, and further, based on molecular docking analysis, 2-methoxy-4-vinylphenold and benzofuran were found to be the major compounds that lead to higher antimicrobial efficacy, due to possessing higher degrees of interaction with DNA gyrase and lipoprotein respectively, of the bacterial cell wall. In this study, the influence of red cabbage extract on the shelf-life of meat under refrigeration storage was also evaluated, showing that the shelf-life of the beef was increased (up to eight days) in terms of microbial and physicochemical properties compared to the control. Therefore, these results demonstrate the effectiveness of red cabbage extract as a natural preservative in the meat processing industry.

Nemati and coworkers [5] have carried out studies on improving the quality characteristics and shelf-life of meat and the growth performance of geese fed diets supplemented with vitamin E. This study was mainly focused on the determination of the effect of diet vitamin E supplementation on growth performance parameters, carcass characteristics, cellular immune system, serum metabolites and shelf-life of goose meat. The obtained results show that the addition of vitamin E in the diet significantly increased the protein and fat content as well as the saturated, monounsaturated and polyunsaturated fatty acids of goose meat, with respect to those obtained from the control diet. Therefore, this study suggests that vitamin E supplementation could be an important alternative for retailers and meat processors to improve the growth performance of geese and the nutritional value of the meat.

The study carried out by Hussain et al. [6] is focused on the effect of gum Cordia extracted from the mature Cordia fruit on pasting, thermal, rheological and texture properties of the corn starch. Gum Cordia is a natural polysaccharide and can be easily extracted from the mature Cordia fruit. The reported results suggest that the incorporation of gum Cordia significantly increased starch gelatinization temperatures, enthalpies and viscosity, as well as the consistency index of the starch–gum blends, which increased with higher levels of gum Cordia. Thus, this study demonstrated that the presence of gum Cordia resulted in a reduction in syneresis that could be due to gum interaction with amylose. These investigations suggest an important alternative of using gum Cordia, as opposed to the other commonly used hydrocolloids, such as xanthan, gum arabic, guar gum, etc., thus improving the cost and safety, and having less time consumption and less requirements than chemical and enzymatic modification.

In the study carried out by Saleh and co-workers [7], it was shown that the olive leaf extract possesses antioxidant and antimicrobial properties. This extract is able to inhibit microbial growth, and therefore increases the shelf-life of the foods to which it is added. Olive leaf extract is rich in polyphenolic compounds. In this work, it was determined that the extract increased the shelf-life of poultry meat, which improves the safety and quality of poultry meat and, on the other hand, reduces the economic losses caused by the decomposition of the meat [7]. The olive extract does not modify the sensory characteristics of the poultry meat. Therefore, olive leaf extract could be used as a natural antioxidant and as a preservative in certain foods.

The study carried out by Mota et al. [8] is also included in this Special Issue. In this work, the authors studied different physical properties of cookies made with different flours enriched with lupine seed protein extract. The addition of this extract to gluten and gluten-free flours showed an improvement in the structure of the dough, where the degree of structuring was increased. Cookies made from buckwheat and oat flour were always firmer than the corresponding control cookies. Regarding the thickness of the cookies, this parameter is important because a lower thickness suggests more crispness, and this property is desirable and highly appreciated by consumers. Cookies made with gluten-free flours (spelt, oat and kamut) and supplemented with lupine seed protein extract produced cookies with a significant reduction in thickness, unlike gluten-free flours (rice and buckwheat wheat flours). Different colorimetric parameters are also determined in the studied cookies, and the authors observed that supplementing the flours with lupine seed protein extract improves color and decreases luminosity, which makes the cookies more appreciated by consumers. On the other hand, when studying parameters related to the conservation of the sample, such as water activity (aw) values and moisture content, the authors determined that all samples have very low values of both parameters, therefore cookies enriched with lupine seed protein are a highly stable food. Therefore, due to all the evaluated parameters, it can be determined that lupine seed protein extract can be used successfully in bakery foods, such as cookies, to obtain a protein-enriched product.

This Special Issue also includes the study by Márquez-Rodríguez et al. [9], in which it was studied if the addition of an extract of *Hibiscus sabdariffa* L., rich in phenolic compounds, could increase the shelf-life of beef meat, due to its antibacterial activity. The authors obtained a phenolic extract from *Hibiscus calyces*, which was fractionated to evaluate the antibacterial activity of the compounds from the different fractions. It was determined that the fraction extracted from the organic phase, which was the richest in phenolic acids, had a higher antimicrobial activity. In the work, the authors evaluated the use as a natural preservative of an ethanolic extract of *Hibiscus sabdariffa* L. rich in phenolic compounds, and for this, the prolongation of the shelf-life of beef meat was determined by adding this extract. In addition, sensory analyses were carried out on the meat. The study determines that adding the phenolic extract of hibiscus to beef meat increases the duration of the shelf-life. Therefore, this extract could be used as a natural preservative for certain foods, as the hibiscus flower is considered a GRAS product (generally recognized as safe). Furthermore, the flavor of the meat sprinkled with the studied extract has a pleasant flavor and the color is similar to that of cooked meat. Therefore, the application of hibiscus extract as an antibacterial marinade for meat could be proposed. This is a healthy option to consume antioxidants and a natural alternative to increase the shelf-life of meat.

The study carried out by Youssef et al. [10] describes the chemical composition of the essential oils from *Chrysanthemum indicum* and *Chrysanthemum morifolium* plants and their possible use as natural preservatives. This study provides evidence for the antimicrobial activity of the essential oils of these plants. The authors studied the composition of the essential oils of *C. indicum* and *C. morifolium* by gas chromatography/flame ionization detector and gas chromatography/mass spectrometry analysis. Camphor is described as the main component of both oils. Essential oils were found to have high antimicrobial, antiviral, anti-mycobacterial, anti-parasitic and antioxidant activity, with *C. indicum* oil being the most effective. Therefore, these oils could be used as spices in food, being able to be incorporated as natural preservatives in different foods and pharmaceutical preparations.

Ahuja et al. [11] included in their study the most recent advances to improve the yield and reduce the cost of xylitol production. This compound exerts different functions and/or activities in the organism that ingests it, that is, it prevents the demineralization of teeth and bones, otitis infection, respiratory tract infections, inflammation and cancer progression. Its applications in different industries, i.e., food, pharmaceutical, cosmetic and polymer, has made it a product with great demand and has made xylitol one of the 12 best bioproducts. The production of xylitol using microorganisms is profitable, but on the other hand, the large-scale application is problematic due to the unstable expression and the non-constant yield. The challenge in the production of this compound can be addressed with the involvement of more advanced tools, such as mutagenesis and genome editing, which lead to the development of strains with higher conversion rates. Furthermore, a good process design with multiple products and cost-effective recovery processes would lead to a reduction in product cost. The implication that materials sciences and nanotechnology could have in improving the yield of this compound should also be highlighted.

This Special Issue also includes the work performed by Krasniewska et al. [12], in which the influence of mixtures of two components of Spanish origanum oil (SOO) with Spanish marjoram oil (SMO) or coriander oil (CO) on anti-listerial activity and sensory quality was studied. In this work, the chemical composition of essential oils is determined by gas chromatography. The results of the study showed that the combined essential oils have a synergistic effect against *L. monocytogenes*. The study showed that essential oil mixtures in sub-inhibitory concentrations inactivate *L. monocytogenes* in vegetable filtrate and in minimally processed vegetables. This study establishes which are the optimal combinations of essential oils based on SOO + SMO to achieve the highest anti-listerial activity in the final products. Furthermore, the authors establish whether the essential oil combinations affect the organoeleptic characteristics and observe that they do not affect the deterioration of the smell or taste.

We are pleased to present this Special Issue, which includes eleven papers that highlight the most important of the research activities in the field of the multiple applications of natural products in foods. With these works, it is intended to advance in the knowledge about how natural compounds could improve certain properties in food. We are very grateful to the authors who have shared their scientific knowledge and experience through their contribution to this Special Issue. We sincerely hope that the readers will find this Special Issue interesting and informative.

## Data Availability

Not applicable.
